# Progress in Fevers

**Published:** 1904-02-27

**Authors:** 


### Progress in Fevers.

(*Continued from page* 375.)

Sleeping Sickness.—The first account of this disease was published rather more than a hundred years ago by Winterbottom.[29] Since then various observers—Carre, Hirsch, Stephen Mackenzie, and Clark—have paid attention to the subject. Manson was one of the first to suggest the disease might be of parasitic origin. In 1897, writing in "Allbutt's System," he suggested the possibility of filaria perstans being the cause of the disease. Since then the discovery of several germs has been announced by different observers. Castellani, working in Uganda, in West Africa, as a member of the British Royal Society Commission sent out to investigate the disease, isolated from the cerebro-spinal fluid of infected persons a micro organism belonging to the streptococcus group and holding a midway position between Fraenkel's diplococcus pneumoniæ and streptococcus pyogenes. This organism he believed to have some causal relation to the disease, and to be different from any organism hitherto described. The members of the Portuguese Commission working in Loanda, in East Africa, four in number—viz. Bettencourt, Kopke, De Rezende, and Mendes—claim, however, that the organism discovered by Castellani appears to be identical in every respect with the micrococcus previously discovered by them in the cerebro-spinal fluid removed *intra vitam* by lumbar puncture, in the blood, and in the spleen, and other organs examined after death. The bacterium was also seen by them in numerous microscopical sections, especially of the nervous tissue. The first report of the Commission, and apparently the only one seen by Castellani, was incomplete and perhaps somewhat misleading in its description, especially of the cultural peculiarities of the coccus; but renewed careful investigations, the results of which were embodied in a *second report* published in Portuguese prior to the publication of Castellani's report, showed the organism in question, called by its discoverers "hypnococcus," to be really identical with the coccus described by Castellani.[30] These investigations of the Portuguese seem to show, at any rate, that the disease is not as strictly limited in its distribution to Equatorial West Africa as has hitherto been supposed. Further researches of Castellani [31] have, moreover, brought further facts to light—viz. the presence in sleeping sickness of trypanosomes in the cerebro-spinal fluid. Combining this discovery with his previous investigations, this observer suggests that a concomitant streptococcal infection plays some part in the disease, at any rate in its later stages. Castellani [32] first observed the presence of trypanosomes in sleeping sickness patients in November, 1902, whilst searching for the micrococcus originally suspected by him to be the causal agent. During the next five months, whilst he remained in Uganda, he found trypanosomes in 20 out of 34 cases examined. His method of procedure was to take 15 ccm. of cerebro-spinal fluid withdrawn by lumbar puncture from a case of sleeping sickness,[33] and to centrifugalise this for 15 minutes. In the resulting white deposit the parasites were readily found on microscopic examination. Following up Castellani's work and using the same methods Bruce and Nabarro, members of the second Royal Commission, have confirmed his discovery and have found trypanosomes in the cerebro-spinal fluid of 40 cases examined. No trypanosomes were found in 15 cases not suffering from sleeping sickness subjected to a similar examination. The report of this second Commission to Uganda [34] is drawn up by Lieutenant-Colonel Bruce and details the work carried on by Nabarro, Greig, and himself in the neighbourhood of Entebbe, Uganda, from March to August, 1903. The Commission adopted Castellani's methods and closely followed his lines of investigation and were thus able to elaborate and confirm his observations as to the presence of trypanosomes in the cerebro-spinal fluid of sufferers from this disease. The report thus summarises its conclusions:—(1) Sleeping sickness is caused by the entrance into the blood and cerebro spinal fluid of a species of trypanosome; (2) this species is probably that discovered by Ford and described by Dutton, from the West Coast of Africa, and called by him trypanosoma gambiense; (3) the so-called cases of trypanosoma fever described from the West Coast may be, and probably are, cases of sleeping sickness in the earliest stages; (4) monkeys are susceptible to the disease, which shows the same symptoms and runs the same course whether the organisms injected are derived from cases of so-called trypanosoma fever, or from the cerebro-spinal fluid of cases of sleeping sickness; (5) dogs and rats are partially susceptible, but guinea pigs, donkeys, oxen, goats, and sheep up to the present have shown themselves absolutely refractory; (6) the trypanosomes are transmitted from the sick to the healthy by a species of tsetse fly, *glossina palpalis*, and by it alone; (7) the distribution of sleeping sickness and *glossina palpalis* correspond; (8) the disease is, in short, a human tsetse-fly disease. Not all of these conclusions have escaped criticism. Sambon [35]

thinks it probable that the glossina does not merely carry the trypanosomes, but that it plays the part of an alternative host. He also points out that although it is pretty certain that glossina does propagate the disease, the experiments carried out by Bruce were not sufficiently exact to prove the point. Manson [36] considers also that the experiments on monkeys, as conducted, cannot be assumed to prove the connection between the two. Although he himself thinks it extremely probable that the trypanosome is the cause of the disease he suggests that caution in accepting this conclusion is advisable since the facts have not yet received absolutely conclusive demonstration. He recalls how a good many years ago he himself discovered filaria perstans in the blood of a sleeping-sickness patient, and on investigating the subject further he obtained a great deal of evidence which seemed to show that there was a causal relationship between the two. Subsequent investigations, however, showed that filaria perstans was merely an accidental inhabitant in the blood of the sufferer. Now he is glad to disallow any connection between the two, and remembers with thankfulness the fact that he never went farther than to suggest that filaria perstans was very likely to be the cause of sleeping sickness. This experience makes him cautious in accepting too hastily the probable truth that trypanosoma gambiense is the cause. The investigations and discoveries of all observers tend ultimately to advance the general well being. Questions of priority in discovery are thorny subjects and should not be too eagerly and jealously discussed. Tsetse flies, as pointed out by M. Brumpt,[37] are found chiefly along the borders of the rivers, in which situations the complaint is most prevalent. A striking instance of this is given. At a mission station worked by Belgian Trappists, at Banamia, a native village 20 minutes' walk from the banks of the Congo, the disease is very rare. The villagers, who are cultivators, drink spring water, and rarely go down to the river side. On the banks of the river, on the other hand, there lived a few years ago some 3,000 Lolo fishermen. So great have the ravages of the sickness been among them, that their numbers are now reduced to 300. The Prime Minister of Uganda [38] regards the exaction of the hut-tax as a fruitful source of the spread of the disease. In lieu of payment of this tax thousands of men proceed yearly to Entebbe, an infected district, and whilst working for the Government for one month live in grass huts along the shore of the lake.

Yellow Fever.—Finlay [39] has made some interesting and ingenious speculations as to the possible nature and life cycle of the yellow fever germ (published in the *Revista de Medicina Tropical*, April 1903). His arguments are based on the difference in duration of the clinical course of yellow fever and malaria. From the analogy between the two diseases the specific germ of yellow fever is expected to prove a protozoon, but whereas the human is the permanent host of the malarial parasite, the mosquito (*Stegomyia*) is regarded as the permanent host of the yellow fever parasite. In malaria the course of events briefly is :—The embryos leave the salivary glands of the mosquito and enter the blood of a non-immune human host, where they become mature and multiply by schizogonia or sporulation, continuing to do so long after they have become ripe for sexual reproduction. The recurrence of sporulation at regular intervals provokes the regularly recurring chills which, when untreated, may recur for months. If these mature spores are drawn into the body of an anopheles, owing to one of these insects biting a malarial human being, they undergo sexual reproduction, with the result that a large number of embryos find their way into the insects' salivary glands ready for migration again into another permanent host. Sexual reproduction takes place only in the body of the mosquito, not in the human body. Should the spores in human blood not find their way into anopheles, there to undergo sexual reproduction, they, after a while, form resting bodies, crescents which remain in the blood of the human host without causing symptoms. Crescents may remain in the blood for months and be capable of undergoing sexual reproduction, if taken into the body of the mosquito. The possible duration of life of the malarial embryos in the mosquito's salivary glands is probably short, as the tiny body of the mosquito could not furnish sufficient pabulum for the embryos to fully develop. In yellow fever, on the other hand, human beings suffering from the disease are only infective to mosquitos during the first few days of illness, that is, according to Finlay's hypothesis, when the organisms are undergoing sexual reproduction in the human blood, during which time they elaborate powerful toxins, to which the attack of yellow fever may be ascribed. It is known that the yellow-fever germ can live in stegomyia for at least two months, and probably until the death of the mosquito. Since the mosquito is so small, Finlay suggests that the organism must be very minute, possibly of ultra-microscopic size. Perhaps also it shares the food in the mosquito's alimentary canal, or it may be parasitic to the bacteria which have been found in association with yellow fever. Resting forms of the organism should, according to this hypothesis, be looked for in the body of the mosquito, not in human infected blood. Certain observers, Parker, Beyer, and others working in Vera Cruz,[40] claim to have discovered and worked out the life history of a protozoon parasite, which may possibly be the causal agent of the disease. They found in the stomach and œsophageal diverticula of mosquitos fed on yellow fever patients small fusiform protozoa arranged simply or more often in groups. These bodies after conjugation develop into sporoblasts, which pass to the salivary glands of the insect, and break up into resting spores. From such infected mosquitos permitted to bite one human being, yellow fever was produced, and the spores were recovered in the bodies of other mosquitos suffered to feed on the blood of this patient. At Vera Cruz, the sanitary and domestic arrangements are such that *stegomyia fasciata* finds abundant natural breeding places and abounds.[41] The larvæ and pupæ will not live in water which is agitated. If a barrel containing them be shaken, they immediately scurry to the bottom. J. B. Tombleson,[42] writing from Island de Tehuantepec, somewhere in the neighbourhood of Mexico describes a bacillus found in his own blood during two mild attacks of yellow fever, and post-mortem in the blood and organs of a fatal case of the fever. The serum of a dog injected with the bacillus readily clumped a pure agar culture, and a monkey injected with a culture died in three days with black vomit and motions, albuminuria, Cheyne-Stokes breathing, and hiccough. The same bacillus was readily recovered from the blood and organs of the animal after death. Subsequently the organism was found in the blood and urine of five other cases. The bacilli stained well with toluidin blue, but poorly or not at all with methylene blue, or carbo-fuchsin. They did not stain well with Gram's method. They stained a bright red after 24 hours' immersion in water, to which a saturated alcoholic solution of hæmatoxylin had been added.

[29] Med. Rec., June 27. [30] Ibid. [31] Lancet, Sept. 12, p. 769. [32] Brit. Med. Jour., Dec. 12, p. 1565. [33] Ibid., Oct. 17, p. 1008. [34] Ibid., Nov. 21. [35] Lancet, Dec. 19, p. 1721. [36] Ibid. [37] Ibid., Aug. 29, p. 636. [38] Ibid., Sept. 12, p. 769. [39] Med. Rec., Aug. 8, p. 217, and Lancet, June 11, p. 1711. [40] Med. Rec., Aug. 15, p. 257. [41] Ibid, Aug. 22, p. 301. [42] Lancet, Aug. 29, p. 594.

*(To be continued.)*

